# Epicardial adipose tissue is associated with cardiorespiratory fitness and hemodynamics among Japanese individuals of various ages and of both sexes

**DOI:** 10.1371/journal.pone.0254733

**Published:** 2021-07-14

**Authors:** Yousuke Sugita, Katsuhiko Ito, Shigeki Sakurai, Satoshi Sakai, Shinya Kuno

**Affiliations:** 1 Faculty of Health Sciences, Tsukuba University of Technology, Kasuga, Tsukuba-shi, Ibaraki, Japan; 2 Graduate School of Comprehensive Human Sciences, University of Tsukuba, Tennoudai, Tsukuba-shi, Ibaraki, Japan; 3 Department of Rehabilitation, Saitama National Hospital, Suwa, Wakoh-shi, Saitama, Japan; 4 Department of Cardiology, Sakurai Clinic, Shibasaki, Takasaki-shi, Gunma, Japan; Scuola Superiore Sant’Anna (SSSA), Pisa and Fondazione Toscana G. Monasterio (FTGM), ITALY

## Abstract

Epicardial adipose tissue may affect hemodynamics and cardiorespiratory fitness as it is a metabolically active visceral adipose tissue and a source of inflammatory bioactive substances that can substantially modulate cardiovascular morphology and function. However, the associations between epicardial adipose tissue and hemodynamics and cardiorespiratory fitness remain unclear. This cross-sectional study aimed to examine the association between epicardial adipose tissue volume and hemodynamics, and cardiorespiratory fitness among Japanese individuals of various ages and of both sexes. Epicardial adipose tissue volume was measured in 120 participants (age, 21–85 years) by cardiac magnetic resonance imaging. To evaluate cardiorespiratory fitness, peak oxygen uptake was measured by cardiopulmonary exercise testing. Peak cardiac output and arteriovenous oxygen difference were calculated by impedance cardiography. The epicardial adipose tissue volume was significantly increased in middle-aged and older women. The epicardial adipose tissue volume was significantly and negatively correlated to peak cardiac output and peak oxygen uptake, regardless of age and sex; furthermore, epicardial adipose tissue showed a strong negative correlation with peak heart rate. Epicardial adipose tissue and peak cardiac output were significantly associated (β = -0.359, 95% confidence interval, -0.119 to -0.049, p < 0.001), even after multivariate adjustment (*R*^*2*^ = 0.778). However, in the multiple regression analysis with peak oxygen uptake as a dependent variable, the epicardial adipose tissue volume was not an independent predictor. These data suggest that increased epicardial adipose tissue volume may be correlated with decreased peak oxygen uptake, which might have mediated the abnormal hemodynamics among Japanese people of various ages and of both sexes. Interventions targeting epicardial adipose tissue could potentially improve hemodynamics and cardiorespiratory fitness.

## Introduction

Cardiorespiratory fitness (CRF) can improve the quality of life and survival of both healthy individuals and patients with cardiovascular disease [1,2]. Considering the consistent association between CRF levels and prognosis [1–3] and health status [4], it is suggested that high CRF levels are effective in preventing lifestyle-related diseases, including cardiovascular disease [5,6]. Thus, it is important to clarify the factors related to CRF across various age groups to prevent cardiovascular disease, improve life prognosis, and maintain quality of life.

Peak oxygen uptake (peakVO_2_) is an index of CRF that is associated with multiple factors, such as aging [7–9], sex [9], daily physical activity [10], and regional adipose tissue [11,12]. Several studies have suggested a substantial association between regional adipose tissue and CRF levels [11,12]. Epicardial adipose tissue (EAT) is an ectopic fat that serves as an index of cardiac and visceral adiposity [13]; it is also a cardiometabolic risk marker [14]. EAT contains abundant cytokines and influences the myocardium and coronary arteries via the paracrine release of cytokines [15]. As a cardiometabolic risk marker, several studies have suggested that EAT may affect peakVO_2_ [16,17] and cardiac function [17,18]. However, these studies did not examine physical activity [19] or the hemodynamic response as a possible mediator of the association between EAT and peakVO_2_. Considering the anatomical location of EAT and its relationship with the resting left ventricular (LV) function, EAT may negatively affect cardiac output (CO) during exercise. There are limited studies investigating the association between the volume of EAT and the hemodynamics response [20,21]. In addition, it is also necessary to examine cardiac function during exercise for both sexes at various ages because the effect of EAT on resting cardiac function differs depending on sex and age [22]. To the best of our knowledge, no study has examined the association between the volume of EAT and the hemodynamic response, including the CO and arteriovenous oxygen difference (a-vO_2_ diff) in various ages and of both sexes.

Thus, we hypothesized that EAT would affect VO_2_ and CO during peak exercise because EAT is a metabolically active visceral adipose tissue (VAT) and a source of inflammatory bioactive substances that can substantially modulate cardiovascular morphology and function. The purpose of this study was to examine the association between EAT volume and hemodynamics, and CRF among Japanese individuals of various ages and of both sexes.

## Materials and methods

### Study design and participants

**Fig 1:**
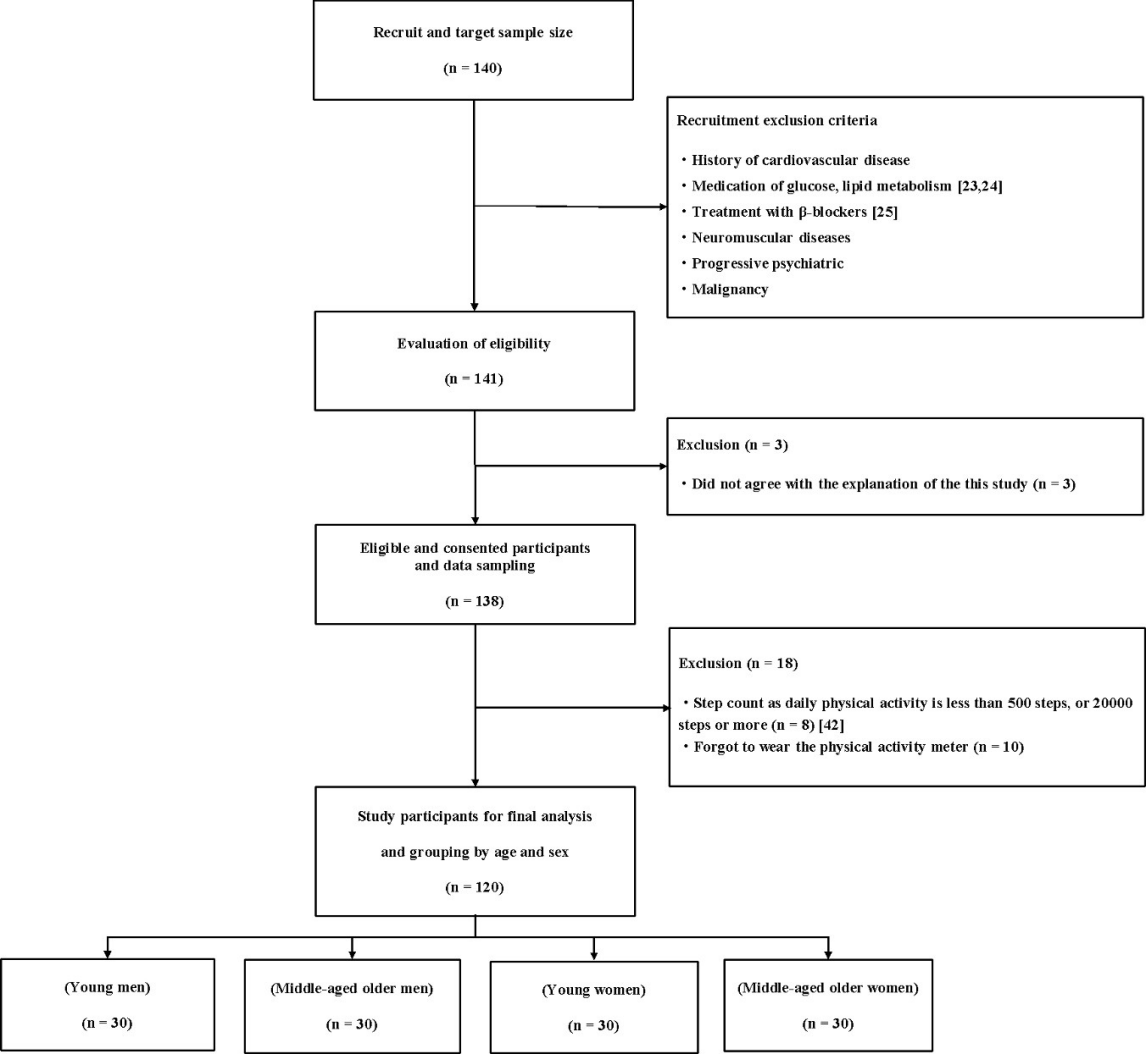
Study design and exclusion criteria. Individuals taking medications for lipid metabolism were excluded because they have been shown to reduce the EAT volume [23,24]. Those taking antihypertensive medications were not excluded because antihypertensive medications have not been reported to affect the EAT volume. Moreover, treatment with β-blockers reduce the heart rate response during exercise; thus, individuals taking such drugs were excluded from the study [25].

This cross-sectional study recruited participants through a public relations magazine. After a meticulous screening procedure, a total of 120 Japanese participants (60 men, 60 women) aged between 21 and 85 years (mean: 51.3 ± 18.7 years) were included in this study. The study participants were divided by age and sex as follows: young (<40 years; 30 men, 30 women) and middle-aged and older (≥40 years; 30 men, 30 women). The inclusion and exclusion criteria for selecting the study participants are shown in Fig 1 [23–25]. The study protocol was explained to all the participants, and informed written consent was obtained from all. This study was conducted in accordance with the Declaration of Helsinki guidelines, and the study protocol was reviewed and approved by the ethics committee (approval number, 30–8) of the Tsukuba University of Technology in Tsukuba City, Japan.

### Measurements of anthropometric parameters, biochemical data, and blood pressure

Body mass index (BMI) and body surface area (BSA) were calculated by measuring height and weight (S1 Table). Obesity and overweight were determined based on the criteria of the World Health Organization of Obesity, with individuals having a BMI from 25.0–29.9 kg/m^2^ being classified as overweight and BMI ≥30.0 kg/m^2^ classified as obese [26]. BSA was calculated following Dubois et al.’s formula [27]. Waist circumference was measured twice with a body tape (WM02; Chamors Ltd. London, United Kingdom), which was placed at the umbilical level of individuals, while they were standing upright. Two measurements were obtained at the end of expiration, and the average value of the two measurements was used.

Blood was drawn from the study participants after 12 hours of fasting and before ingesting hypoglycemic agents. Ten milliliters of blood was collected and stored in a blood collection tube (VenojectII; Terumo Co., Ltd., Tokyo, Japan). The tube remained at room temperature for 15 min and was then placed in an automatic analyzer (LABOSPECT008; Hitachi High Technologies Co., Ltd., Tokyo, Japan). After undergoing centrifugation for 10 min, the plasma and blood cell components were separated. Triglycerides, total cholesterol, high-density lipoprotein cholesterol, and low-density lipoprotein cholesterol were measured according to the methods described in S1 Table.

The homeostasis model assessment of insulin resistance, a surrogate measure of insulin resistance, is a simple index based on the fasting plasma glucose and insulin levels in a fasting blood sample [28]. The formula for calculating homeostasis model assessment of insulin resistance is shown in S1 Table. Hemoglobin A1c levels were determined by high-performance liquid chromatography and expressed using the National Glycohemoglobin Standardization Program unit.

Systolic and diastolic blood pressure were measured from the arms of seated participants, after a 20-min rest, using an automatic blood pressure monitor (HEM-7220, Omron Healthcare Co., Ltd. Kyoto, Japan). Before using the blood pressure monitor, its accuracy was checked with a mercury sphygmomanometer. Hypertension, hyperglycemia, and dyslipidemia were determined according to the Japanese Diagnosis Criteria (S1 Table) [29].

### Measurement of EAT volume

For EAT volume, all participants underwent 1.5 Tesla nuclear magnetic resonance imaging (MRI) (Ingenia; PHILIPS-Japan Co., Ltd. Tokyo, Japan), as described in previous studies (S1 Table) [30,31]. The EAT was finally determined by multiplying the number of fat voxels within the three-dimensional regions of interest by the voxel size and was normalized to the BSA (S1 Table) [30].

### Measurements of abdominal visceral and subcutaneous adipose tissues

The VAT and subcutaneous adipose tissue were imaged by abdominal MRI (S1 Table).

### Measurement of cardiac function by MRI

Cardiac function in the supine position was measured by cardiac MRI. The values of LV end systolic volume, LV end diastolic volume, LV ejection fraction, and LV mass (LVM) were determined offline by tracing the endocardial border manually [32]. Stroke volume index (SI), cardiac output index (CI), and LV ejection fraction were calculated according to the methods described in S1 Table. LVM was additionally normalized to the BSA for the LVM index. Based on a previous study [33], the LV hypertrophy (LVH) was defined as a LVM index of >96 g/m^2^ for men and >82 g/m^2^ for women.

### Measurement of CRF and hemodynamic response

The CRF was measured by cardiopulmonary exercise testing (CPET) with a symptomatic limit using an ergometer (232C-XL; Combi Co., Ltd., Tokyo, Japan). PeakVO_2_ [34] and anaerobic threshold [35] were measured according to the methods described in S1 Table. In addition, metabolic equivalents (METs), heart rate recovery (HRR), and peakVO_2_/heart rate (HR) were calculated using the method described in S1 Table. The predicted peakVO_2_ [36] and anaerobic threshold [36] were determined according to previous studies (S1 Table).

The hemodynamic response from the sitting position to peak exercise was measured using a non-invasive transthoracic bioimpedance device (PhysioFlow PF-05 Lab1; Manatec Biomedical, Paris, France) during the CPET. With regards to the measurement of CO using PhysioFlow, a high correlation with the direct Fick method has been clarified in healthy individuals and those with illnesses [37,38]. The measurement items in the PhysioFlow were SV and HR. The CO and a-vO_2_ diff were calculated using the method described in S1 Table. The chronotropic incompetence was determined to be <80% of the predicted maximum HR according to the report of Laforgia et al. [39]. Based on the report of Cole et al., an abnormal value for the HRR was defined as a reduction of 12 beats/min or less from the heart rate at peak exercise [40].

### Measurement of physical activity

Daily physical activity was estimated from the magnitude and frequency of the acceleration signal detected at 32 Hz using a pedometer with a multiple memory accelerometer (Lifecorder; SUZUKEN CO., LTD. Nagoya, Japan) and the movement related calorie consumption to the physical activity and the number of steps (S1 Table). Validity of the Lifecorder has been confirmed in comparison with that of metabolic chambers and the dual-labeled water method [41]. We assumed that a step count value for >20,000 steps/day, and <500 steps/day are not a routine step count value [42].

### Measurement of lower limb muscle strength performance

The 30-s chair-stand test (CS-30) for evaluating lower limb muscle strength was measured according to the method [43] described in S1 Table.

### Statistical analysis

Normally distributed data are expressed as mean ± standard deviation, whereas non-normally distributed data are expressed as median. SPSS version 24 (IBM Japan, Ltd. Tokyo, Japan) was used for all statistical analyses. Using a two-tailed test, the significance level was set to <5%. No logarithmic transformation was performed on the data. Outliers were not excluded because there were no medically apparent unusual values, and the robustness was maintained with no difference between the analyses with and without data that appeared to be outliers. However, if there was even one missing data point (i.e., physical activity measurement, etc.), it was excluded from the analysis. The sample size was estimated to be 120 participants by calculation, assuming that the significance level (α) is 0.05, power (1-β) is 0.8, the effect size is 20, and the standard deviation is 27, based on previous studies on EAT volume. For data analysis, we tested the normality using the Shapiro–Wilk test. The χ^2^ and Fisher’s exact tests were performed for nominal scale data, and Harberman’s residual analysis was performed on the adjusted residual value. The unpaired Student’s t-test and Mann-Whitney U test were used to compare the differences in the data between sexes and age groups. Furthermore, age- and sex-adjusted multiple regression analyses were used to compare the EAT volume according to the presence or absence of cardiovascular disease risk factors and exercise intolerance (S2 Table). Next, Spearman’s rank correlation coefficient, Pearson’s correlation coefficient, and partial correlation analysis, with BMI, fasting plasma glucose, and left ventricular mass index (LVMI) as control variables, were used to determine the relationship between EAT volume and peakVO_2_, METs, peak SI, peak HR, HRR, and peak CI. Stepwise method multiple linear regression analysis was performed to determine the variables independently associated with the peakVO_2_ and peak CI in Japanese individuals of various ages. First, a multiple linear regression analysis with peakVO_2_ as the dependent variable was performed, whereas the age [7–9], sex [9], BMI [44], steps of daily physical activity [10], presence of hypertension [45], presence of hyperglycemia [46], presence of dyslipidemia [47], peak stroke volume index [48], peak heart rate [48], visceral adipose tissue [49], and epicardial adipose tissue volume were used as the independent variables. Second, a multiple linear regression analysis with peak CI as the dependent variable was performed, while the independent variables included the age, sex, BMI, steps of daily physical activity, presence of hypertension, presence of hyperglycemia, presence of dyslipidemia, LVMI, VAT, and EAT volume. We referred to the report of Pugliese et al. [50] and selected the independent variables that are known to be important determinants for peakVO_2_ and peak CI. The normality of distribution of the residuals in the multiple regression analysis (Tables 4 and S2) was confirmed by the Shapiro-Wilk test for residuals. In addition, there were no variables with a correlation coefficient of ≥0.8 or a variance inflation factor of ≥5.0 among the independent variables.

## Results

### Selection of participants and clinical characteristics

Of the total 138 applicants, 120 individuals were enrolled in this study of which 41.7% were overweight, 25.8% were current smokers, 64.2% had hypertension, 13.3% had dyslipidemia, and 13.3% had hyperglycemia.

### EAT volume measurements and its characteristics

The EAT volume was significantly greater in the participants with metabolic syndrome components (i.e., hypertension, hyperglycemia, and dyslipidemia), LVH, physical inactivity (movement related calorie consumption <300 kcal), abnormal value for the HRR (abnormal value for the HRR ≦12beats from the HR at peak exercise), and exercise intolerance (predicted peakVO_2_ and predicted anaerobic threshold <80%) than in those without such factors, However, there was no significant difference in the EAT volume between current smokers and non-smokers (S2 Table).

### Clinical characteristics by age and sex

The BSA-normalized EAT volume showed the highest value in the middle-aged or older female group, followed by the middle-aged or older male group, the young female group, and the young male group. In the middle-aged or older female group, low-density lipoprotein cholesterol level, triglycerides level, and the prevalence of dyslipidemia and LVH were significantly higher than those of the middle-aged or older male group. Prevalence of overweight, daily physical activity, and CS-30 score were significantly lower in the middle-aged or older female group than in the middle-aged or older male group. The BSA, VAT, subcutaneous adipose tissue, hemoglobin A1c, daily physical activity, and CS-30 score were significantly lower in the young female group than in the young male group (Table 1).

**Table 1:** Characteristics of the participants classified by age and sex.

	Male		Female	
Characteristics	Young	Middle-aged/older	Young	Middle-aged/older
**N**	30	30	30	30
**EAT (mL/m**^**2**^**)**	50.9 (IQR 49.1–56.0)	65.8 (IQR 59.8–74.5)*	52.4 (IQR 47.6–58.1)	81.3 (IQR 68.9–87.2)*^,^^†^
**Age (years)**	34 (IQR 30–38)	71 (IQR 66–74)*	37 (IQR 32–38)	67 (IQR 63–71)*
**Anthropometric parameters**				
** Height (cm)**	167 (IQR 163–171)	168 (IQR 160–169)	153 (IQR 150–158)^†^	153 (IQR 151–157)^†^
** Weight (kg)**	69.5 (IQR 64.8–71.4)	66.4 (IQR 61.2–71.0)	57.1 (IQR 54.6–71.5)^†^	55.1 (52.0–58.8)^†^
** Body mass index (kg/m**^**2**^**)**	24.9 (IQR 23.8–25.8)	24.0 (IQR 22.7–25.2)	24.7 (IQR 23.6–25.8)	23.7 (IQR 22.1–25.1)*
** Body surface area (m**^**2**^**)**	1.78 (IQR 1.71–1.82)	1.75 (IQR 1.67–1.81)	1.53 (IQR 1.50–1.61)^†^	1.53 (IQR 1.48–1.58)^†^
** Waist circumference (cm)**	101.5 (IQR 98.5–103.3)	102.0 (IQR 101.0–104.0)	98.5 (IQR 93.3–103.8)	102.0 (IQR 101.0–106.3)*
** VAT (cm**^**2**^**)**	92.5 (IQR 88.8–94.3)	103.0 (95.3–107.0)*	82.5 (IQR 75.0–89.0)^†^	103.0 (IQR 101.0–107.0)*
** SAT (cm**^**2**^**)**	120.1 ± 10.9 (103–139)	130.2 ± 10.3 (111–149)*	139.4 ± 11.2 (120–150)^†^	129.6 ± 10.3 (110–151)*
** Overweight (%)**	50	46.7	40	30
** Obesity (%)**	0	0	0	0
**Physical activity and lower limb muscle strength**				
** Steps (steps/days)**	6917 ± 1234 (3604–10091)	4949 ± 864 (2489–6612)*	5730 ± 1416 (3931–9235)^†^	4398 ± 1544 (2287–7884)*
** Movement related to calorie consumption (kcal/days)**	347 (IQR 300–394)	229 (IQR 185–299)*	256 (IQR 223–309)^†^	176 (IQR 139–222)*^,^^†^
** 30-Second chair-stand test (times/30seconds)**	27 (IQR 24–33)	22 (IQR 15–26)*	18 (IQR 15–19)^†^	11 (IQR 8–17)*^,^^†^
**Preference and medication**				
** Smoker (%)**	23	30*	23.3	26.7
** ACEI (%)**	0	26.7*	0	36.7*
** ARB (%)**	0	63.3*	20^†^	53.3*
** CCB (%)**	0	23.3*	13	30
** Diuretic (%)**	0	13.3	3	20
** ARB + CCB (%)**	0	23.3*	10	30
** ARB + diuretic (%)**	0	13.3*	3.3	20
** ARB + CCB + diuretic (%)**	0	3.3	3	13.3
**Biochemical analysis and blood pressure**				
** Total cholesterol (mg/dL)**	201.2 (IQR 168.3–207.3)	217.3 (IQR 208.5–227.1)*	204.2 (IQR 181.3–212.1)	228.7 (IQR 212.0–241.3)*^,^^†^
** LDL cholesterol (mg/dL)**	106.2 ± 22.5 (68.2–138.4)	127.5 ± 27.4 (56.6–187.6)*	113.6 ± 25.9 (64.4–158.6)	144.6 ± 27.9 (86.6–194.6)*^,^^†^
** HDL cholesterol (mg/dL)**	61.7 ± 13.9 (39.4–97.3)	60.3 ± 13.6 (35.1–82.2)	62.2 ± 13.3 (44.3–96.4)	54.2 ± 13.9 (29.3–83.3)*
** Triglyceride (mg/dL)**	105.9 (IQR 99.1–118.7)	131.2 (IQR 119.0–142.2)*	112.2 (IQR 99.8–134.5)	140.2 (IQR 124.5–143.8)*
** Fasting plasma glucose (mg/dL)**	90.1 ± 5.7 (81.3–101.2)	99.4 ± 6.7 (86.2–112.2)*	92.2 ± 7.4 (79.3–108.3)	101.3 ± 8.1 (87.2–119.2)*
** Fasting plasma insulin (μU/mL)**	6.1 (IQR 5.3–6.2)	8.2 (IQR 6.2–9.2)*	6.2 (IQR 5.3–7.2)	8.2 (IQR 6.5–9.3)*
** Hemoglobin A1c (%)**	5.1 (IQR 4.8–5.3)	5.2 (IQR 5.0–5.4)*	5.3 (IQR 5.2–5.4)^†^	5.4 (IQR 5.3–5.4)*
** HOMA-IR (%)**	1.3 (IQR 1.1–1.4)	2.1 (IQR 1.5–2.3)*	1.4 (IQR 1.2–1.7)	2.1 (IQR 1.6–2.5)*
** Systolic blood pressure (mmHg)**	126 (IQR 122–128)	128 (IQR 124–142)*	138 (IQR 123–146)^†^	128 (IQR 124–142)
** Diastolic blood pressure (mmHg)**	72 (IQR 70–80)	78 (IQR 68–84)	76 (IQR 69–85)	78 (IQR 68–84)
** Hypertension (%)**	36.7	93.3*	36.7	90*
** Hyperglycemia (%)**	3.3	16.7	3.3	30*
** Dyslipidemia (%)**	6.7	10	6.7	30*
**Cardiac function in supine position measured by MRI**				
** Left ventricular end-diastolic volume (mL)**	101.3 (IQR 94.5–112.0)	99.2 (IQR 95.5–104.2)	99.8 (IQR 90.5–101.9)	90.3 (IQR 84.1–101.3)*^,^^†^
** Left ventricular end-systolic volume (mL)**	27.2 (IQR 25.4–33.6)	27.7 (IQR 25.8–30.3)	30.3 (IQR 28.5–31.9)	29.8 (IQR 25.8–30.3)
** Left ventricular ejection fraction (%)**	71.3 ± 4.7 (61.7–80.0)	71.0 ± 5.4 (53.9–76.7)*	68.6 ± 4.5 (58.9–78.2)^†^	68.2 ± 4.9 (56.9–79.7)^†^
** SI (mL/m**^**2**^**)**	41.6 ± 6.1 (29.1–53.3)	42.4 ± 6.5 (28.7–57.9)	41.2 ± 6.6 (30.6–56.7)	41.2 ± 7.2 (32.1–61.6)
** HR (bpm/min)**	68 (IQR 66–72)	68 (IQR 66–73)	68 (IQR 66–72)	69 (IQR 67–74)
** CI (L/min/m**^**2**^**)**	1.6 (IQR 1.5–2.0)	1.6 (IQR 1.4–1.8)	1.9 (IQR 1.6–2.2)	1.9 (1.6–2.1)^†^
** Left ventricular mass index (g/m**^**2**^**)**	83.9 (IQR 69.2–102.6)	96.8 (IQR 78.2–111.4)	81.6 (71.5–96.1)	95.4 (86.4–114.2)*
** Presence of left ventricular hypertrophy (%)**	36.7	53.3	36.7	83.3*^,^^†^

The normally distributed data are expressed as mean ± standard deviation, whereas the non-normally distributed data are expressed as a median, and the nominal variables are expressed as percentages

*P < 0.05 vs same-sex young group

^†^P < 0.05 vs men in each corresponding age group.

Abbreviations: EAT: Epicardial adipose tissue, VAT: Visceral adipose tissue, SAT: Subcutaneous adipose tissue, ACEI: Angiotensin-converting enzyme inhibitor, ARB: Angiotensin receptor blocker, CCB: Calcium channel blocker, LDL: Low-density lipoprotein, HDL: High-density lipoprotein, HOMA-IR: Homeostasis model assessment of insulin resistance, MRI: Magnetic resonance imaging, SI: Stroke volume index, HR: Heart rate, CI: Cardiac output index.

### Exercise performance and hemodynamic response by age and sex

With regard to the CPET data, the mean peak respiratory exchange ratio was >1.15 in all groups and no-load shortage was observed. Approximately 42.5% of the participants in the middle-aged or older group reported that the reasons for terminating the exercise load were related to the pedal rotation speed of the exercise bike falling below 50 rotations. The highest peakVO_2_ value was observed in the young male group, followed by the young female group, the middle-aged or older male group, and the middle-aged or older female group. The highest value of the peak CI was also observed in the young male group, followed by the young female group, the middle-aged or older male group, and the middle-aged or older female group. In the middle-aged or older female group, the prevalence of chronotropic incompetence was higher than that of the middle-aged or older male group. The peak HR, predicted peak HR, and predicted anaerobic threshold were significantly lower in the middle-aged or older female group than in the middle-aged or older male group. The peak SI and peak HR were significantly lower in the young female group than in the young male group (Table 2).

**Table 2:** Cardiopulmonary exercise testing and hemodynamics of participants classified by age and sex.

	Male		Female	
Characteristics	Young	Middle-aged or older	Young	Middle-aged or older
**Resting (sitting posture)**				
** RER**	0.82 (IQR 0.81–0.83)	0.83 (IQR 0.82–0.83)	0.83 (IQR 0.82–0.84)	0.83 (IQR 0.81–0.83)
** VO**_**2**_ **(mL/min/kg)**	3.4 (IQR 3.2–3.5)	3.3 (IQR 3.1–3.5)	3.6 (IQR 3.4–3.8)^†^	3.5 (IQR 3.1–3.9)
** METs**	1.0 (IQR 0.9–1.0)	0.9 (IQR 0.9–1.0)	1.0 (IQR 1.0–1.1)	1.0 (IQR 0.9–1.1)*
** SI (mL/m**^**2**^**)**	37.2 ± 3.2 (31.9–42.3)	37.1 ± 3.4 (29.5–43.9)	40.3 ± 3.7 (30.5–46.8)^†^	37.3 ± 3.4 (28.6–42.9)*
** HR (bpm/min)**	72 (IQR 70–75)	72 (IQR 69–72)	71 (IQR 69–72)	71 (IQR 68–72)
** CI (L/min/m**^**2**^**)**	2.7 ± 0.3 (2.3–3.5)	2.7 ± 0.3 (2.1–3.5)	2.9 ± 0.3 (2.1–3.3)^†^	2.6 ± 0.3 (1.9–3.1)*
** a-vO**_**2**_ **diff (mL/100mL)**	5.0 (IQR 4.5–5.3)	4.8 (IQR 4.1–5.2)	4.7 (IQR 4.2–4.9)	4.8 (4.8–5.3)*
** VO**_**2**_**/HR (mL/beat)**	3.2 (IQR 3.0–3.4)	3.0 (IQR 2.8–3.1)	3.1 (IQR 2.7–3.2)^†^	2.8 (IQR 2.6–3.0)^†^
**Anaerobic threshold**				
** RER**	0.99 (IQR 0.98–1.01)	0.99 (IQR 0.99–1.01)	0.99 (IQR 0.98–1.01)	1.00 (IQR 0.98–1.00)
** VO**_**2**_ **(mL/min/kg)**	20.6 (IQR 18.1–25.3)	12.5 (IQR 11.1–15.2)*	17.8 (IQR 15.6–20.0)	11.2 (IQR 9.2–13.4)*^,^^†^
** METs**	5.9 (IQR 5.2–7.2)	3.6 (IQR 3.2–3.4)*	5.1 (IQR 4.5–5.7)	3.2 (IQR 2.6–3.8)*^,^^†^
** Work rate (watt)**	148 (IQR 118–176)	76 (IQR 65–92)*	101 (IQR 88–136)^†^	50 (IQR 42–64)*^,^^†^
** SI (mL/m**^**2**^**)**	66.3 ± 4.8 (58.1–76.4)	58.9 ± 5.4 (48.3–72.4)*	58.2 ± 5.3 (50.8–71.1)^†^	54.6 ± 7.2 (42.9–77.3)*^,^^†^
** HR (bpm/min)**	140 (IQR 124–164)	99 (IQR 93–109)*	135 (131–152)	91 (84–102)*^,^^†^
** CI (L/min/m**^**2**^**)**	9.3 (IQR 8.0–11.5)	5.8 (IQR 5.4–6.8)*	7.9 (IQR 7.2–8.9)^†^	4.8 (4.0–6.3)*^,^^†^
** a-vO**_**2**_ **diff (mL/100mL)**	8.6 ± 0.7 (6.2–10.2)	8.2 ± 0.9 (6.4–10.1)	8.6 ± 0.9 (7.4–10.5)	8.2 ± 0.6 (7.2–9.4)
** VO**_**2**_**/HR (mL/beat)**	10.3 (IQR 9.4–10.8)	8.2 (IQR 7.7–9.1)*	7.6 (IQR 6.9–9.0)^†^	6.8 (IQR 5.9–7.5)*^,^^†^
**Peak exercise**				
** RER**	1.16 (IQR 1.15–1.18)	1.16 (IQR 1.16–1.18)	1.16 (IQR 1.15–1.18)	1.17 (IQR 1.15–1.17)
** VO**_**2**_ **(mL/min/kg)**	32.2 (IQR 27.6–37.9)	18.1 (IQR 14.7–21.5)*	26.2 (19.9–30.5)^†^	15.2 (IQR 12.7–21.3)*
** METs**	9.2 (IQR 7.9–10.8)	5.2 (IQR 4.2–6.1)*	7.5 (IQR 5.7–8.7)^†^	4.3 (IQR 3.6–6.1)*
** Work rate (Watt)**	206 (IQR 164–250)	106 (IQR 88–136)*	148 (IQR 101–189)^†^	75 (IQR 57–106)*^,^^†^
** SI (mL/m**^**2**^**)**	69.1 ± 5.6 (59.6–79.5)	58.0 ± 6.5 (45.4–75.5)*	62.1 ± 6.1 (52.7–75.8)^†^	56.1 ± 7.6 (43.7–74.6)*
** HR (bpm/min)**	179 (IQR 158–208)	124 (IQR 116–137)*	162 (IQR 142–192)	116 (IQR 106–130)*^,^^†^
** CI (L/min/m**^**2**^**)**	12.8 (IQR 10.6–15.1)	7.3 (IQR 6.5–8.0)*	10.2 (IQR 8.6–12.0)^†^	6.1 (IQR 5.2–8.2)*^,^^†^
** a-vO**_**2**_ **diff (mL/100mL)**	9.9 (IQR 9.6–10.0)	9.6 (IQR 9.0–10.2)	9.6 (IQR 9.0–10.6)	9.5 (IQR 8.7–10.2)
** VO**_**2**_**/HR (mL/beat)**	11.9 ± 1.4 (7.9–14.4)	9.7 ± 1.7 (7.2–14.2)*	9.5 ± 1.7 (7.0–12.9)^†^	8.1 ± 1.8 (5.0–11.1)*^,^^†^
**Other indicators**				
** Oxygen uptake efficiency slope**	2318 (IQR 1954–2889)	1313 (IQR 1133–1657)*	1646 (IQR 1168–1939)^†^	892 (IQR 715–1150)*^,^^†^
** Percent of peak HR (%)**	96.1 (IQR 86.5–111.0)	80.0 (IQR 78.6–91.8)*	88.0 (IQR 78.1–103.8)	74.7 (IQR 70.6–84.8)*^,^^†^
** Chronotropic incompetence (%)**	16.7	46.7*	30	63.3*
** Heart rate recovery (beat)**	16 (IQR 16–24)	11 (IQR 9–14)*	16 (IQR 16–26)	9 (IQR 7–11)*^,^^†^
**Abnormality of heart rate recovery (%)**	3.3	66.7*	13.3	86.7*
**Reason for end of exercise load**				
** Leveling off of VO**_**2**_	0	0	0	0
** SBP decreased by 10 mmHg with exercise load, and SBP was ≥250 mmHg**	3.3	6.7	3.3	10.0
** RPE**_**R**_ **and RPE**_**L**_ **>17**	26.7	30.0	13.3	33.3
** Pedal speed of <50 rpm**	36.7	43.3	43.3	46.7
** Request for termination from study participants**	33.3	20	40	10*
** Percent of peakVO**_**2**_ **(%)**	80.6 (IQR 70.9–92.8)	72.1 (IQR 58.8–90.3)	80.6 (IQR 62.9–95.0)	62.6 (IQR 54.3–81.8)*
** Percent of anaerobic threshold (%)**	83.1 (IQR 73.4–96.5)	75.2 (IQR 65.1–94.9)	78.3 (IQR 72.0–90.5)	63.3 (IQR 56.7–79.0)*^,^^†^

The normally distributed data are expressed as mean ± standard deviation, whereas the non-normally distributed data are expressed as median, and nominal variables are expressed as percentages

*P < 0.05 vs same-sex young group

^†^P < 0.05 vs men in each corresponding age group.

%peak HR was calculated as follows: Peak HR/220 –age × 100).

Chronotropic incompetence was defined as <80% of the predicted maximum HR by age.

Heart rate recovery was calculated as follows: Peak HR– 1 min after the cessation of exercise [40].

Abnormal value for the heart rate recovery was defined as a reduction of ≦12beats from the heart rate at peak exercise [40].

Percent of peakVO_2_ and anaerobic threshold were determined based on a previous study [36].

Abbreviations: RER: Respiratory exchange ratio, IQR: Interquartile range, VO_2_: Oxygen uptake, METs: Metabolic equivalents, SI: Stroke volume index, HR: Heart rate, CI: Cardiac output index, a-vO_2_ diff: Arterial-venous oxygen difference, VO_2_/HR: O_2_ pulse, SBP: Systolic blood pressure, RPE_R_: Rating of perceived exertion on the respiratory, RPE_L_: Rating of perceived exertion on the lower extremity.

### Correlation between EAT volume and peakVO_2_ and hemodynamics

In all participants, EAT volume was negatively correlated with peakVO_2_ and peak CI. In the young groups, EAT was significantly correlated with peakVO_2_ and peak CI, and the degree of simple correlation was approximately the same between men and women. EAT was also significantly correlated with peakVO_2_ and peak CI in the middle-aged or older group, but it was more tightly correlated with the middle-aged or older female group than with the middle-aged or older male group. In all participants, the results of the partial correlation analysis, using BMI, fasting plasma glucose, and LVMI as control variables, showed a significant correlation between EAT volume and peakVO_2_, METs, peak SI, peak HR, HRR, and peak CI (Table 3).

**Table 3:** Correlation between regional adipose tissue and hemodynamics.

	All participants		Men				Women			
			Young		Middle-aged and older		Young		Middle-aged and older	
	Simple correlation	Partial correlation	Simple correlation	Partial correlation	Simple correlation	Partial correlation	Simple correlation	Partial correlation	Simple correlation	Partial correlation
**PeakVO**_**2**_ **(mL/min/kg)**										
** EAT (mL/m**^**2**^**)**	-0.867*	-0.592*	-0.827*	-0.559*	-0.684*	-0.615*	-0.789*	-0.641*	-0.881*	-0.678*
** VAT (cm**^**2**^**)**	- 0.587*	-0.428*	-0.130	-0.398^†^	0.021	-0.041	-0.852*	-0.802*	-0.499^†^	-0.177
** SAT (cm**^**2**^**)**	- 0.061	-0.169	-0.083	-0.297	-0.043	-0.168	0.085	0.024	0.300	0.039
**METs (peak exercise)**										
**EAT (mL/m**^**2**^**)**	-0.867*	-0.593*	-0.785*	-0.557*	-0.677*	-0.611*	-0.791*	-0.643*	-0.881*	-0.686*
**VAT (cm**^**2**^**)**	-0.588*	-0.429*	-0.191	-0.397^†^	0.017	-0.043	-0.850*	-0.803*	-0.509*	-0.179
**SAT (cm**^**2**^**)**	-0.022	-0.170	-0.080	-0.291	0.062	-0.169	0.106	0.020	0.197	0.038
**Peak CI (L/min/m**^**2**^**)**										
** EAT (mL/m**^**2**^**)**	-0.894*	-0.582*	-0.796*	-0.480^†^	-0.645*	-0.631*	-0.836*	-0.650*	-0.879*	-0.685*
** VAT (cm**^**2**^**)**	-0.636*	-0.396*	-0.055	-0.328	-0.098	-0.099	-0.925*	-0.845*	-0.502^†^	-0.157
** SAT (cm**^**2**^**)**	-0.121	-0.259^†^	-0.127	-0.353	-0.032	-0.151	0.050	-0.029	0.227	-0.094
**Peak SI(mL/min/m**^**2**^**)**										
** EAT (mL/m**^**2**^**)**	-0.653*	-0.375*	-0.353	0.175	-0.296	-0.233	-0.434^†^	-0.240	-0.740*	-0.495*
** VAT (cm**^**2**^**)**	-0.462*	-0.159	0.129	0.067	-0.060	-0.069	-0.659*	-0.534*	-0.418^†^	-0.205
** SAT (cm**^**2**^**)**	-0.119	-0.219^†^	-0.014	-0.055	-0.150	-0.221	0.209	0.130	0.224	-0.062
**Peak HR (bpm/min)**										
** EAT (mL/m**^**2**^**)**	-0.931*	-0.658*	-0.868*	-0.669*	-0.641*	-0.668*	-0.910*	-0.795^†^	-0.927*	-0.752*
** VAT (cm**^**2**^**)**	-0.669*	-0.480*	-0.144	-0.398^†^	-0.081	-0.081	-0.916*	-0.852^†^	-0.550^†^	-0.070
** SAT (cm**^**2**^**)**	-0.080	-0.198^†^	-0.141	-0.374	-0.074	-0.056	-0.019	-0.085	0.185	-0.150
**HRR (bpm)**										
**EAT (mL/m**^**2**^**)**	-0.932*	-0.651*	-0.807*	-0.459^†^	-0.680*	-0.646*	-0.955*	-0.828^†^	-0.813*	-0.753*
**VAT (cm**^**2**^**)**	-0.662*	-0.564*	-0.055	-0.255	0.213	0.036	-0.817*	-0.724^†^	-0.660*	-0.355
**SAT (cm**^**2**^**)**	0.036	-0.072	-0.197	-0.418^†^	0.064	-0.136	0.080	0.011	0.258	-0.019

*P < 0.001

^†^P < 0.05. Partial correlation analysis was performed with the BMI, fasting plasma glucose, and left ventricular mass index as control variables. Abbreviations: PeakVO_2_: Peak oxygen uptake, METs: Metabolic equivalents, EAT: Epicardial adipose tissue, VAT: Visceral adipose tissue, SAT: Subcutaneous adipose tissue, CI: Cardiac output index, SI: Stroke volume index, HR: Heart rate, HRR: Heart rate recovery.

### Independent factors associated with peakVO_2_ and peak CI

In the multiple linear regression analysis with the peakVO_2_ as the dependent variable, the age, sex, BMI, steps as physical activity, peak SI, and peak HR were found to be the independent factors associated with the peakVO_2_ in all participants (*R*^*2*^ = 0.954). Whereas in the multiple regression analysis with the peak CI as the dependent variable, the age, sex, presence of hypertension, LVMI, and EAT (β = -0.359, 95% confidence interval = -0.119 to -0.049, p < 0.001) were found to be the independent factors associated with the peak CI (*R*^*2*^ = 0.779) (Table 4).

**Table 4:** Multiple linear regression analysis with peakVO_2_ and peak CI as dependent variables.

Independent variable	PeakVO_2_				Peak CI			
	R^2^	SE	Standardized β	P-value	R^2^	SE	Standardized β	P-value
	**0.954**				**0.779**			
**Age**		**0.017**	**0.125**	**<0.001**		**0.013**	**-0.341**	**<0.001**
**Sex (female)**		**0.418**	**-0.073**	**0.001**		**0.324**	**0.161**	**<0.001**
**Body mass index**		**0.103**	**-0.072**	**0.001**				
**Steps (physical activity)**		**0.000**	**0.097**	**<0.001**				
**Presence of hypertension**						**0.413**	**-0.214**	**<0.001**
**Peak stroke volume**		**0.035**	**0.325**	**<0.001**				
**Peak heart rate**		**0.009**	**0.779**	**<0.001**				
**Left ventricular mass index**						**0.009**	**-0.113**	**0.043**
**Epicardial adipose tissue**						**0.018**	**-0.359**	**<0.001**

Multiple linear regression analysis was performed using the stepwise method; the dependent variables were peakVO_2,_ and peak CI. We referred to the report of Pugliese et al. [50] and selected the independent variables that are known to be important determinants for peakVO_2_ and peak CI. To confirm multicollinearity between the independent variables, a correlation coefficient of ≥0.8 or a variance inflation factor of ≥5.0 was looked for, but neither was confirmed. In addition, on performing the Shapiro-Wilk test on residuals, the significance probability was each 0.098 and 0.066, thus confirming their normal distributions.

Abbreviations: PeakVO_2_: Peak oxygen uptake, peak CI: Peak cardiac output index, SE: Standard error.

## Discussion

This cross-sectional study clarified the relevance of EAT and hemodynamics, and CRF, including the physical activity among Japanese individuals of various ages and of both sexes. In the present study, we observed three major findings. First, the EAT volume measured by MRI was negatively correlated with peak CI, a cardiac function index during submaximal exercise in all participants. Second, EAT volume was negatively correlated with peakVO_2_ in all participants. Third, EAT volume was independently associated with peak CI among Japanese individuals of various ages and of both sexes, even when the multivariate analyses were adjusted. However, the EAT volume was not shown as a peakVO_2_ predictor when the peak SI and peak HR, which are components of the Fick’s equation, were adjusted. These results suggest that increased EAT volume may be correlated with decreased peakVO_2_, which might have mediated the abnormal hemodynamics among Japanese people of various ages and of both sexes.

Contrary to our hypothesis, the EAT volume was eliminated from the peakVO_2_ predictors after multivariate adjustment. As shown in Table 4, cardiac function indicators, such as the peak HR and peak SI are strong predictors of peakVO_2_, and this result is almost in agreement with the study results of Pugliese et al. [50] and the Fick’s equation. However, as shown in Table 3, the correlation coefficient between EAT volume and peakVO_2_ had a strong correlation of -0.867, suggesting that it might be one of the related factors. Therefore, the relationship between EAT and peakVO_2_ requires further evaluation not only in cross-sectional studies but also in longitudinal or interventional studies.

Several studies have provided clear evidence of a negative association between EAT and peakVO_2_ [16,17,20]. These reports support some of our findings. Kim et al. reported that the EAT thickness, as measured by echocardiography, was independently associated with peakVO_2_ [16]. Despite reporting an association between EAT thickness and peakVO_2_, their study primarily focused on overweight or obese men (mean age 49.0 ± 1.0 years) with a mean BMI of 29.4 kg/m^2^ and did not include women or individuals of different ages. In addition, Sugita et al. reported that the independent association between EAT and peakVO_2_, including the resting LV structure and function [17]. However, they did not examine physical activity or hemodynamic response as a potential mediator of the association between EAT and peakVO_2_. Our present study extended these findings by demonstrating an correlation between EAT and peakVO_2_ and the relationship between EAT and the hemodynamics response that underlies that relationship in Japanese individuals of various ages and of both sexes. However, our findings were in contrast to one study that showed a paradoxical inverse relationship between EAT and peakVO_2_ [49]. The reason for the discrepancy with our study is unclear. The report by Haykowsky et al. [49] targeted older patients with heart failure with preserved ejection fraction and healthy controls without metabolic disorders, such as hypertension and diabetes, while our study targeted variable ages of both sexes. The discrepancies in these results among previous studies may be partially explained by the differences in study participants’ age group, sex, and prevalence of cardiovascular disease. A study by Koepp et al. showed that the inverse relationship observed between EAT and peakVO_2_ may be related to less typical group differences [20].

As shown in Table 3, EAT volume was correlated with peakVO_2_. This correlation might have partially occurred due to the association of EAT volume with the peak CI, which was one of the factors that defined peakVO_2_ (Fick’s equation). The peak CI is the product of SV and HR during peak exercise and is one of the determinants of peakVO_2_, as shown in Fick’s equation. In this study, the EAT volume and peak SI and peak HR were statistically significantly associated. However, to the best of our knowledge, there is no report in previous studies of the EAT excess directly depressing the SV. In addition, as shown in Table 1, LVH and hypertension prevalence tended to increase with EAT volume. Furthermore, as shown in Table 3, the high correlation between EAT volume and peak SI observed in the simple correlation analysis (r = −0.653, p< 0.001) is weakened in the partial correlation analysis (r = −0.375, p< 0.001). When patients with heart valve disease are excluded, as in our study, hypertension and LVH can be indicators of afterload. Increased afterload can be a major hemodynamic factor that reduces SV. Therefore, it is necessary to further study the relationship between EAT and SV.

Partial correlation analysis indicated that the peak HR represents the HR response during exercise, and it was strongly associated with EAT volume (r = −0.658, p< 0.001) in the partial correlation analysis (Table 3). As shown in Table 2, the resting HR was not significantly different between the young and middle-aged and older groups, but the HR during peak exercise was significantly lower in the middle-aged and older group than in the young group. This finding indicated that, in the middle-aged and older people with a high EAT volume, the HR response was poor as the exercise load increased (i.e., peak HR flattened, CI). In fact, as shown in Table 2, the prevalence of chronotropic incompetence was significantly higher in the middle-aged and older group with high EAT volume. The SV reached a plateau at 40–50% of peak exercise, and subsequently, the increasing HR led to CO increase [51]. Furthermore, the cardiac sympathetic nervous system (i.e., HR response) is involved in the exercise load at 60% after peak exercise [52]. Balcioğlu et al. [53] examined 224 individuals without heart disease and reported that the cardiac autonomic function parameters, such as HR variability and HR turbulence, were significantly worsened in the group of high EAT volume with a median EAT thickness of ≥3.9 mm. This study partially supports our findings. However, although the HR response was not found as one of the factors of exercise intolerance, it cannot be concluded as an exercise intolerance determinant because the present study had a cross-sectional design. Furthermore, in the middle-aged and older group, approximately 45% of the patients reported that the reason for ending the CPET was decreased pedal speed, and the CS-30 score was also lower in this group; thus, exercise intolerance due to physical inactivity and lower limb muscle weakness cannot be denied. Thus, further investigation is required to determine the association between EAT volume and HR response and peakVO_2_.

As shown in Table 4, EAT volume was independently associated with peak CI even after multivariable adjustment in all participants. However, in the participants grouped according to age and sex, the EAT volume was the highest and peak CI was the lowest in the middle-aged and older female group which indicated a strong negative correlation. Several studies [54,55] reported that the older female group had significantly lower CO than the younger and older male groups. Although there are many unclear points about the cause of sex differences in CO during peak exercise, Wheatley et al. [55] reported that women’s SV increase slows down with increasing exercise intensity. However, the results of this report are different from our results (Table 2), which showed that the middle-aged and older group had a significant difference in peak HR rather than in SV during peak exercise. It is speculated that this difference from the previous study was due to the difference in the characteristics of the study participants. Our middle-aged and older group was mostly composed of individuals with diseases (i.e., hyperglycemia, hypertension, and LVH), which increases their risk for CI and increase afterload, whereas the study population of Wheatley et al. was composed of healthy individuals. It is inferred that the difference in these attributes is one of the reasons for the difference in the results.

In the present study, the EAT volume was correlated with peakVO_2_ and peak CI in the Japanese individuals of various ages and of both sexes. However, there are several limitations in this study. First, since this study is a cross-sectional study and not a longitudinal or interventional study, it is not possible to prove the causal relationship between EAT accumulation and peakVO_2_ and peak CI as a potential mechanism. Longitudinal and intervention studies are necessary to determine the relationship between the EAT volume and the CO, HR response, and VO_2_. Second, although this study targets Japanese people of various ages and of both sexes, a selection bias cannot be completely denied because it is a single-center study. In addition, our study results are limited to Japanese individuals. It differs from Westerners in terms of race and physique. Therefore, it should be noted that the results are of a different race than Westerners. Third, impedance cardiography is a non-invasive method for assessing CO and has been reported to be highly correlated with the direct Fick method in healthy individuals. However, it has been reported that SV may be overestimated when patients with heart failure with reduced EF are set as participants [56]. Therefore, measurement errors may have occurred in the middle-aged and older group with a high prevalence of LVH. However, our study participants have preserved LV ejection fraction compared to that of the previous study; patients with ischemic and dilated cardiomyopathy were not included, and their clinical characteristics are significantly different. CPET and echocardiography combined stress tests show clinically acceptable measurement accuracy consistent with the CO value measured by direct Fick during exercise. Furthermore, a wide range of information can be obtained (e.g., global longitudinal strain, E/e’, LVEF, and SV). This may provide a compatible alternative to the invasive direct Fick method [57,58]. Fourth, our study did not collect data on cardiac biomarkers or circulating cytokines of the brain natriuretic peptide. However, it has been suggested that EAT may release inflammatory cytokines [59]. These adipokines play an important role in heart disease such as coronary artery disease. It may have been possible to deepen our understanding of crosstalk between EAT and myocardium by collecting data such as inflammatory markers and brain natriuretic peptide. Finally, although cardiac function analysis by cardiac MRI has become the gold standard in terms of non-invasiveness, reproducibility, and accuracy, it is costly and time consuming, which limits its use in routine practice. In clinical practice, echocardiography is often the standard primary technique and is used as one of the common means of follow-up. A comparative measurement using echocardiography and cardiac MRI shows a strong correlation in LVEF measurements, E/e’ as diastolic function, and EAT volume [60–62]. This strong correlation suggests that the data obtained by cardiac MRI in this study may be reproduced with high accuracy even in echocardiography.

Our data suggest that reducing EAT volume, a localized fat close to the heart, may have benefits in hemodynamics and CRF. Certainly, calorie restriction has been shown to improve not only EAT volume but also cardiac function [63]. However, further longitudinal studies are needed to determine whether a decrease in EAT volume is associated with improved hemodynamics and exercise capacity.

## Conclusions

The results of this cross-sectional study involving Japanese people of various ages and of both sexes suggest that the EAT volume may be tightly correlated with peakVO_2_ and peak CI. EAT volume was eliminated from the peakVO_2_ predictors in the results of multivariate analysis along with important confounding factors, such as the hemodynamics and daily physical activity. However, it was still significantly and independently associated with peak CI, which is a component of Fick’s equations. These data suggest that EAT volume may negatively affect hemodynamics and CRF across Japanese individuals of various ages and of both sexes. Interventions targeting EAT could potentially improve hemodynamics and CRF. In the future, it will be necessary to carry out longitudinal studies conducted jointly across multiple institutions to clarify the relationship between the EAT volume and peakVO_2_ and peak CI.

## Supporting information

S1 Table Measurements of anthropometric parameters, biochemical data, blood pressure, epicardial adipose tissue volume, abdominal visceral adipose tissue and subcutaneous adipose tissue, cardiorespiratory fitness, hemodynamics response, physical activity, and lower limb muscle strength. EAT: Epicardial adipose tissue, CRF: Cardiorespiratory fitness.
(DOCX)

S2 Table Difference in the epicardial adipose tissue volume among patients classified according to the presence of cardiovascular disease risk factors and exercise intolerance. Data are represented as mean ± SD, *P< 0.05 vs patients without cardiovascular disease risk factors and exercise intolerance Multiple regression analysis was adjusted for age and sex. Hypertension, hyperglycemia, and dyslipidemia were determined according to the Japanese Diagnosis Criteria [29]. EAT: Epicardial adipose tissue, VAT: Visceral adipose tissue, peak VO_2_: Peak oxygen uptake, SD: Standard deviation.
(DOCX)

S1 Data (SAV)
