# Supplementary material for: Epicardial adipose tissue is associated with cardiorespiratory fitness and hemodynamics among Japanese individuals of various ages and of both sexes
Source: PLoS One. 2021 Jul 14;16(7):e0254733. doi: 10.1371/journal.pone.0254733 (PMC8279356; doi:10.1371/journal.pone.0254733)
Supplement: S1 Table — EAT: Epicardial adipose tissue, CRF: Cardiorespiratory fitness. (DOCX) [file pone.0254733.s001.docx]

**S1 Table**

BMI (kg/m^2^) = body weight (kg) / height^2^ (m)

BSA (m^2^) = body weight^0.425^ × height^0.725^ × 0.007184 [27].

HOMA-IR = fasting insulin (μU/mL) × fasting glucose (mg/dL)/405 [28].

SV (mL) = LVEDV (mL) – LVESV (mL)

LVEF (%) = SV / LVEDV (mL) ×100

Predicted peakVO_2_ (%) = -0.38 × age + 52.1 (For male); -0.23 × age + 40.4 (For female) [36].

Predicted AT (%) = -0.22 × age + 32.3 (For male); -0.16 × age + 27.8 (For female) [36]

VO_2_/HR (mL/beat) = VO_2_ (mL) / HR (beat/min)

CO (L/min) = SV (mL/min) × HR (beat/min)

a-vO_2_ (mL/100 mL) = CO (L) / VO_2_ (mL)

BMI: body mass index, BSA: body surface area, HOMA-IR: homeostasis model assessment of insulin resistance, VO_2_: oxygen uptake, AT: anaerobic threshold, CO: cardiac output, SV: stroke volume, HR: heart rate, a-vO_2_ diff: arteriovenous oxygen difference

**Materials and Methods**

**Measurements of anthropometric parameters, biochemical data, and blood pressure**

Body height was measured to the nearest 0.1 cm using a wall-mounted stadiometer (DC250; Tanita Co. Ltd., Tokyo, Japan). Body weight of barefoot patients was measured to the nearest 0.1 kg using calibrated electronic digital scales (DC250; Tanita).

Triglyceride (TG), total cholesterol, and high-density lipoprotein cholesterol (HDL-C) levels were measured using an enzymatic method. An enzyme solution (Qualijent TG and Choletest N HDL; Sekisui Medical Co., Ltd., Tokyo, Japan) was added to 2–3 μL of the sample. The mixture was heated at 37 °C for 5 min, and absorbance was determined for defining the concentration. In addition, low-density lipoprotein cholesterol (LDL-C) was measured using the direct method. An enzyme solution (Choletest LDL; Sekisui Medical Co., Ltd.) was added to 3 μL of the sample and mixed. The mixture was heated at 37 °C for 5 min, and absorbance was determined for defining the concentration.

Hypertension, hyperglycemia, and dyslipidemia were determined according to the Japanese Diagnosis Criteria [29] as follows: (1) systolic blood pressure (SBP) of ≧130 mm Hg and/ or diastolic blood pressure of ≧85 mm Hg; (2) Fasting plasma glucose levels of ≧110 mg/dL; (3) TG levels of ≧150 mg/dL; (4) HDL-C levels of <40 mg/dL. The diagnostic criteria included those on medication for hypertension, hyperglycemia, and dyslipidemia as additional criteria.

**Measurement of epicardial adipose tissue volume**

A 32-channel trunk coil with a 1.5 Tesla nuclear magnetic resonance imaging (MRI, Ingenia; PHILIPS-Japan Co. Ltd., Tokyo, Japan) apparatus with a maximum gradient strength of 45 mT/m and a maximum slew rate of 120 mT/m/ms was used for signal reception. Electrocardiogram (ECG)-gated steady-state free precession-cine images were obtained while the subjects held their breath in the horizontal long axis, the vertical long axis, left ventricular outflow tract, and short axis for wall motion and functional analysis. The sequence parameters were as follows: field of view (FOV) = 350×350 mm², slice thickness = 8 mm, pixel size = 1.7×1.7 mm^2^ reconstructed to 1×1 mm^2^, repetition time (TR) = 3.1 ms, echo time (TE) = 1.6 ms, flip angle (α) = 60°, parallel imaging factor (SENSE) = 2.5, and number of cardiac phases reconstructed = 40. For assessment of the epicardial adipose tissue (EAT), a three-dimensional (3D) transverse ECG-triggered and respiratory navigator-gated magnetization-prepared mDixon sequence was acquired. Trigger delay was set to the end of diastole and optimized by means of cine MRI data. FOV = 350×302×180 mm^3^, voxel size = 1.5×1.5×3.0 mm^3^ (120 over contiguous slices), reconstructed voxel size = 1.0×1.0×1.5 mm^3^, TR = 5.4 ms, TE1/TE2 = 1.8 ms/4.0 ms; α = 20°, SENSE = 1.5 in both phase encoding directions, water fat shift = 0.16 pixels, and arrhythmia rejection were applied, along with a T2 preparation = 50 ms and acquisition window = 100–156 ms (selected based on cine MRI data). The net scan duration was 3–5 min. With an assumed navigator efficiency of 40–50%, the average total scan duration time was about 7.5 min．In-phase (IP), opposed-phase (OP), water only (W), and fat only (F) images were reconstructed online at the scanner console [30]. Dixon images were analyzed offline on a personal computer using a dedicated software written in MATLAB (MathWorks, Inc. Massachusetts, United States) with an analysis time of about 7–10 minutes per subject. EAT was measured between the bifurcation of the pulmonary artery and the most inferior transverse slice of the myocardium [31]. A 3D region of interest was defined by manually contouring the epicardial border in each slice. The EAT was finally determined by multiplying the number of fat voxels inside the 3D regions of interest by the voxel size and was normalized to the BSA [30].

**Measurements of abdominal visceral adipose tissue and subcutaneous adipose tissue**

Visceral adipose tissue and subcutaneous adipose tissue were photographed using an abdominal MRI. Participants were subjected to a whole-body coil in the supine position and were photographed to obtain a 10-mm thick tomographic image over 10 cm, including umbilicus, by TR: 600 ms，TE: 18 ms，FOV: 350 mm × 350 mm by the spin echo (SE) method．To calculate the abdominal fat area，the image was transferred to another terminal via digital imaging and communications in medicine using a tomographic image of a height passing through the umbilicus. The ROI was set in the fat area to obtain the mean value and standard deviation of the density. Next, considering that the value obtained by doubling the standard deviation corresponded to the lower and upper limit value of the fat area, the portion included within the range was determined by binarization processing. In the binarized image, regions corresponding to visceral adipose tissue and subcutaneous adipose tissue were divided, and the area of each region was analyzed using image analysis software, Image J Ver. 1.33u (National Institute of Health, <http://rsb.info.nih.gov/ij/>)

**Measurement of CRF and hemodynamics response**

The peakVO_2_ and anaerobic threshold (AT) were determined using the ramp loading method, with an increase of 10 watts per minute after a rest period of 4 minutes and warm-up at 0 watts for 4 minutes. The highest oxygen uptake achieved after more than 30 seconds of exercise was determined as the peakVO_2_.

Tanaka et al. [34] used the peakVO_2_ as a criterion. AT was determined using the V-slope method described by Beaver et al. [35].

For measuring expiratory gas, a respiratory analyzer (AE300S; Minato Medical Science Co., Ltd. Tokyo, Japan) was used in the breath-by-breath mode. Exhaled gas data were analyzed using the average of 10 respiratory cycles.

The number of revolutions of the pedal during bicycle movement was 60 revolutions per minute (rpm). HR was constantly monitored at rest, during exercise, and during the recovery period by using an ECG monitor (ML4500; Fukudadenshi Co., Ltd. Tokyo, Japan). The end points of the bicycle movement were as follows: (1) leveling off of VO_2_, (2) decrease in SBP by 10 mmHg with the exercise load and SBP of 250 mmHg or more, (3) rate of perceived exertion about respiratory (RPE_R_) and RPE about lower extremity (RPE_L_) >17, (4) a respiratory exchange ratio (RER) of >1.15, and (5) a pedal speed of <50 rpm (3 seconds or more). If at least one of these 5 conditions was met, it was considered to be completed.

**Measurement of physical activity**

For objective assessment of physical activity, movement-related calorie consumption during physical activity and the number of steps taken were measured continuously for a week by a Lifecorder (SUZUKEN CO., LTD. Nagoya, Japan) worn on the lumbar region of study participants. Data was accepted only when 75% or more of Lifecorder was attached per unit time and was rejected when 25% or more of defects were confirmed due to non-attachment. The defect criterion was as follows: continuous non-wearing for 3 hours or more out of 12 hours (body movement level 0 was regarded as non-wearing).

**Measurement of lower limb muscle strength performance**

The CS-30 was measured using a chair with a height of 40 cm, and the inspector took care to prevent the risk of falling. At the start of the test, the posture was unified so that both lower limbs were spread out to the width of the shoulders, the back was separated from the backrest, and both arms were folded in front of the chest. The measurement was performed only once after several exercises. The standing and sitting motions were repeated as many times as possible in 30 seconds, and the hip and knee joints were to be straightened in the standing position.
