# Supplementary material for: Epicardial adipose tissue is associated with cardiorespiratory fitness and hemodynamics among Japanese individuals of various ages and of both sexes
Source: PLoS One. 2021 Jul 14;16(7):e0254733. doi: 10.1371/journal.pone.0254733 (PMC8279356; doi:10.1371/journal.pone.0254733)
Supplement: S2 Table — Data are represented as mean ± SD, *P< 0.05 vs patients without cardiovascular disease risk factors and exercise intolerance Multiple regression analysis was adjusted for age and sex. Hypertension, hyperglycemia, and dyslipidemia were determined according to the Japanese Diagnosis Criteria [29]. EAT: Epicardial adipose tissue, VAT: Visceral adipose tissue, peak VO2: Peak oxygen uptake, SD: Standard deviation. (DOCX) [file pone.0254733.s002.docx]

S2 Table.

|  | EAT volume (mL/m^2^) | |
| --- | --- | --- |
|  | Absence | Presence |
| Overweight | 65.7 ± 15.5 | 57.2 ± 12.0 ^*^ |
| Current smoker | 62.1 ± 14.7 | 62.3 ± 15.0 |
| VAT >100 cm^2^ | 54.9 ± 10.4 | 74.0 ± 12.9 ^*^ |
| Hypertension | 52.0 ± 11.4 | 67.9 ± 13.2 ^*^ |
| Hyperglycemia | 59.6 ± 13.2 | 79.1 ± 13.1 ^*^ |
| Dyslipidemia | 60.5 ± 14.3 | 73.0 ± 13.1 ^*^ |
| Left ventricular hypertrophy | 52.5 ± 8.6 | 71.0 ± 13.6 ^*^ |
| Physical inactive (MCC <300 kcal) | 51.6 ± 9.2 | 67.1 ± 14.2 ^*^ |
| Percent of peakVO_2_ <80% | 52.4 ± 9.2 | 69.4 ± 13.9 ^*^ |
| Percent of anaerobic threshold < 80% | 51.9 ± 8.8 | 69.0 ± 13.9 ^*^ |
| Heart rate recovery ≦12 bpm | 52.0 ± 6.9 | 76.0 ± 10.6 ^*^ |
| Peak VO_2_/heart rate ≦10 mL/beat | 51.9 ± 7.4 | 71.5 ± 13.4 |

Data are represented as mean ± standard deviation, *P < 0.05 vs patients without cardiovascular disease risk factors and exercise intolerance Multiple regression analysis was adjusted for age and sex. Hypertension, hyperglycemia, and dyslipidemia were determined according to the Japanese Diagnosis Criteria [29]. To confirm multicollinearity between the independent variables, a correlation coefficient of ≥0.8 or a variance inflation factor of ≥5 was looked for but was not confirmed in all items. In addition, on performing the Shapiro-Wilk test on the residuals of the multiple regression analyses, all p-values were ≥0.05, confirming the normality of the residuals.

Abbreviations: EAT: epicardial adipose tissue, MCC: Movement related to calorie consumption VAT: visceral adipose tissue, peakVO_2_: peak oxygen uptake.
